# Health and nutritional status of children hospitalized during the COVID-19 pandemic, Bangladesh

**DOI:** 10.2471/BLT.21.285579

**Published:** 2021-10-20

**Authors:** Sharika Nuzhat, SM Tafsir Hasan, Parag Palit, Farzana Afroze, Rukaeya Amin, Md Ashraful Alam, Baharul Alam, Mohammod J Chisti, Tahmeed Ahmed

**Affiliations:** a Nutrition and Clinical Services Division, International Centre for Diarrhoeal Disease Research, Bangladesh, 68 Shaheed Tajuddin Ahmed Sarani, Mohakhali, Dhaka 1212, Bangladesh.

## Abstract

**Objective:**

To compare the health and nutrition of children younger than 5 years admitted to hospital during and before the coronavirus disease 2019 (COVID-19) pandemic in Bangladesh.

**Methods:**

We collected data from hospital records of children 0–59 months admitted to the Dhaka Hospital of the International Centre for Diarrhoeal Disease Research, Bangladesh in March 2020–February 2021 (COVID-19 period; *n* = 2552) and March 2019–February 2020 (pre-COVID-19 period; *n* = 6738). Data collected included sociodemographic, anthropometric, clinical and biochemical characteristics. We compared these data for child admissions in the COVID-19 and pre-COVID-19 periods, including infants 0–11 months born during and before the pandemic and admitted to hospital.

**Findings:**

Admissions of children as a percentage of total admissions were lower in March 2020 (2.47%; 63/2552) than March 2019 (8.30%; 559/6738), but increased to 20.61% (526/2552) in February 2021, three times greater than in the pre-COVID-19 period (6.69%; 451/6738). Children admitted during the COVID-19 period were significantly more likely to have dehydration, severe sepsis or septic shock, convulsions, hypernatraemia and raised creatinine than children admitted before the pandemic (*P* < 0.05). In infants < 6 months and those born during the pandemic, stunting and wasting were significantly higher than in infants in the pre-COVID-19 period (*P* < 0.05). The risk of death was higher in infants < 6 months during the pandemic (odds ratio: 1.66; 95% confidence interval: 0.95–2.92).

**Conclusion:**

During the pandemic, children presented with more severe illness and poorer nutrition. Efforts are needed to reduce the adverse effects of the pandemic on the health and well-being of children.

## Résumé

**Objectif:**

Comparer l'état de santé et de nutrition des enfants de moins de 5 ans hospitalisés avant et pendant la pandémie de maladie à coronavirus 2019 (COVID-19) au Bangladesh.

**Méthodes:**

Nous avons récolté des données provenant des dossiers d'hospitalisation d'enfants âgés de 0 à 59 mois admis au Centre international de recherche sur les maladies diarrhéiques à Dhaka, au Bangladesh, entre mars 2020 et février 2021 (période COVID-19; *n* = 2552) ainsi qu'entre mars 2019 et février 2020 (période pré-COVID-19; *n* = 6738). Les données collectées reprenaient les caractéristiques sociodémographiques, anthropométriques, cliniques et biochimiques. Nous avons ensuite comparé ces données relatives aux admissions d'enfants au cours des périodes pré-COVID-19 et COVID-19, en y incluant les nourrissons de 0 à 11 mois nés avant et pendant la pandémie puis hospitalisés.

**Résultats:**

Le pourcentage d'enfants sur le nombre total d'admissions était moins important en mars 2020 (2,47%; 63/2552) qu'en mars 2019 (8,30%; 559/6738). En revanche, il est passé à 20,61% (526/2552) en février 2021, un chiffre trois fois plus élevé que durant la période pré-COVID-19 (6,69%; 451/6738). Les enfants hospitalisés au cours de la pandémie de COVID-19 étaient nettement plus susceptibles de souffrir de déshydratation, d'une septicémie sévère ou d'un choc septique, de convulsions, d'hypernatrémie et d'un taux de créatinine accru que les enfants hospitalisés avant la pandémie (*P* < 0,05). L'émaciation et le retard de croissance étaient beaucoup plus fréquemment observés chez les nourrissons de moins de 6 mois et ceux nés durant la pandémie de COVID-19 que chez les nourrissons ayant vu le jour avant (*P* < 0,05). Le risque de décès était plus élevé chez les nourrissons de moins de 6 mois durant la pandémie (rapport de cotes: 1,66; intervalles de confiance de 95%, 0,95-2,92).

**Conclusion:**

La pandémie a entraîné une recrudescence des maladies graves et un appauvrissement nutritionnel chez les enfants. Des efforts sont nécessaires pour atténuer l'impact négatif de la pandémie sur leur santé et leur bien-être.

## Resumen

**Objetivo:**

Comparar la salud y la nutrición de los niños menores de 5 años ingresados en el hospital durante y antes de la pandemia de la coronavirosis de 2019 (COVID-19) en Bangladesh.

**Métodos:**

Se recopilaron datos de los registros hospitalarios de niños de entre 0 y 59 meses admitidos en el hospital de Dhaka del Centro Internacional de Investigación de Enfermedades Diarreicas, Bangladesh, entre marzo de 2020 y febrero de 2021 (periodo de la COVID-19; *n*= 2552) y entre marzo de 2019 y febrero de 2020 (periodo pre-COVID-19; *n*= 6738). Los datos recopilados incluyeron características sociodemográficas, antropométricas, clínicas y bioquímicas. Se compararon estos datos para los ingresos de niños en los periodos de la COVID-19 y pre-COVID-19, incluidos los bebés de entre 0 y 11 meses nacidos durante y antes de la pandemia e ingresados en el hospital.

**Resultados:**

Los ingresos de los niños como porcentaje del total de ingresos fueron menores en marzo de 2020 (2,47 %; 63/2552) que en marzo de 2019 (8,30 %; 559/6738), pero aumentaron al 20,61 % (526/2552) en febrero de 2021, tres veces más que en el periodo pre-COVID-19 (6,69 %; 451/6738). Los niños ingresados durante el periodo de la COVID-19 tuvieron una probabilidad mucho mayor de presentar deshidratación, sepsis grave o choque séptico, convulsiones, hipernatremia y elevación de la creatinina que los niños ingresados antes de la pandemia (*P* <0,05). En los lactantes <6 meses y en los nacidos durante la pandemia, el retraso en el crecimiento estatural y la emaciación fueron muy superiores a los de los lactantes en el periodo pre-COVID-19 (*P* <0,05). El riesgo de muerte fue mayor en los lactantes <6 meses durante la pandemia (oportunidad relativa: 1,66; intervalos de confianza del 95 %: 0,95-2,92).

**Conclusión:**

Durante la pandemia, los niños presentaron enfermedades más graves y una peor nutrición. Es preciso esforzarse por reducir los efectos indeseables de la pandemia en la salud y el bienestar de los niños.

## ملخص

الغرض مقارنة صحة وتغذية الأطفال الذين تقل أعمارهم عن 5 سنوات، والذين يخضعون للعلاج بالمستشفى أثناء جائحة مرض فيروس كورونا 2019 وبعدها (كوفيد 19) في بنغلاديش.

**الطريقة:**

قمنا بجمع البيانات من سجلات مستشفيات الأطفال منذ الولادة وحتى عمر 59 شهرًا الذين دخلوا مستشفى دكا التابع للمركز الدولي لأبحاث أمراض الإسهال، في بنغلاديش خلال الفترة من مارس/آذار 2020 إلى فبراير/شباط 2021 (فترة كوفيد 19؛ العدد = 2552)، والفترة من مارس/آذار 2019 إلى فبراير/شباط 2020 (فترة ما قبل كوفيد 19؛ العدد = 6738). تضمنت البيانات التي تم جمعها الخصائص الاجتماعية السكانية، والأنثروبومترية، والإكلينيكية، والكيميائية الحيوية. قمنا بمقارنة هذه البيانات الخاصة بالتحاق الأطفال بالمستشفى في فترة كوفيد 19، وفترة ما قبل كوفيد 19، بما في ذلك الرضع منذ الولادة وحتى عمر 11 شهرًا والذين ولدوا أثناء الجائحة وقبلها، والتحقوا بالمستشفى.

**النتائج:**

كان التحاق الأطفال بالمستشفى كنسبة مئوية من إجمالي الالتحاقات، أقل في مارس/آذار 2020 (2.47%؛ 63/2552) عنه في مارس/آذار 2019 (8.30%؛ 559/6738)، لكنه ارتفع إلى 20.61% (526/2552) في فبراير/شباط 2021، إلى أكثر من ثلاثة أمثال ما كانت عليه في فترة ما قبل كوفيد 19 (6.69%؛ 451/6738). كان الأطفال الملتحقون بالمستشفى خلال فترة كوفيد 19 أكثر عرضة بشكل ملموس للإصابة بالجفاف، والإنتان الشديد أو الصدمة الإنتانية، والتشنجات، وفرط صوديوم الدم، وزيادة الكرياتينين مقارنة بالأطفال الملتحقين بالمستشفى قبل الجائحة (نسبة الاحتمال أقل من 0.05). في الرضع الأقل من 6 أشهر، وأولئك الذين ولدوا أثناء الجائحة، كان التقزم والهزال أعلى بشكل ملموس في الرضع في فترة ما قبل كوفيد 19. (نسبة الاحتمال أقل من 0.05). كان خطر الوفاة أعلى عند الرضع الأقل من 6 أشهر أثناء الجائحة (نسبة الأرجحية: 1.66؛ فواصل الثقة 95%: 0.95 إلى 2.92).

**الاستنتاج:**

خلال الجائحة، كان الأطفال يعانون من أمراض أكثر شدة وتغذية أكثر سوءًا. هناك حاجة إلى بذل جهود للحد من الآثار السلبية للجائحة على صحة الأطفال ورفاهيتهم.

## 摘要

**目的:**

旨在比较孟加拉国新型冠状病毒肺炎 (COVID-19) 大流行期间和大流行之前 5 岁以下住院儿童的健康和营养状况。

**方法:**

我们从 2020 年 3 月至 2021 年 2 月（新型冠状病毒肺炎大流行期间；n = 2552）和 2019 年 3 月至 2020 年 2 月（新型冠状病毒肺炎大流行之前的时期；n = 6738）在孟加拉国国际腹泻病研究中心达卡医院收治的 0-59 个月儿童的医院记录中收集数据。收集的数据包括社会人口学、人体测量学、临床和生物化学方面的数据。我们比较了新型冠状病毒肺炎大流行期间和大流行之前住院儿童的这些数据，包括大流行期间和大流行之前出生并住院的 0-11 个月大的婴儿。

**结果:**

2020 年 3 月的儿童住院率（2.47%；63/2552）比 2019 年 3 月（8.30%；559/6738）低，但在 2021 年 2 月增加到 20.61%（526/2552），是新型冠状病毒肺炎大流行之前 (6.69%；451/6738) 的三倍。在新型冠状病毒肺炎大流行期间住院的儿童比大流行前（P < 0.05）住院的儿童更容易出现脱水、严重败血症或感染性休克、抽搐、高钠血症和肌酐升高的症状。在不到 6 个月大的婴儿和大流行期间出生的婴儿中，出现发育迟缓和消瘦的婴儿明显高于新型冠状病毒肺炎大流行之前（P < 0.05）的婴儿。在大流行期间，不到 6 个月大的婴儿的死亡风险更高（优势比：1.66；95% 置信区间：0.95-2.92）。

**结论:**

大流行期间，儿童患有更严重的疾病和更加营养不良。需要努力减少大流行对儿童健康的不利影响。

## Резюме

**Цель:**

Сравнить состояние здоровья и питания детей младше 5 лет, поступивших в больницу во время пандемии и до пандемии коронавирусной инфекции в 2019 году (COVID-19) в Бангладеш.

**Методы:**

Авторы собрали данные из историй болезни детей в возрасте 0–59 месяцев, поступивших в больницу Международного центра исследований диарейных заболеваний, г. Дакка, Бангладеш, в периоды с марта 2020 г. по февраль 2021 г. (период COVID-19; n = 2552) и с марта 2019 г. по февраль 2020 г. (период до COVID-19; n = 6738). Собранные данные включали социально-демографические, антропометрические, клинические и биохимические характеристики. Мы сравнили эти данные по госпитализации детей в период COVID-19 и период до COVID-19, включая младенцев в возрасте 0–11 месяцев, родившихся и госпитализированных во время пандемии и до пандемии.

**Результаты:**

Объемы госпитализации детей в процентном отношении к общему числу поступивших в больницу в марте 2020 г. были ниже (2,47%; 63/2552), чем в марте 2019 г. (8,30%; 559/6738), но увеличились до 20,61% (526/2552) в феврале 2021 г., что в три раза больше, чем в период до COVID-19 (6,69%; 451/6738). У детей, госпитализированных в период COVID-19, вероятность обезвоживания, тяжелого сепсиса или септического шока, судорог, гипернатриемии и повышения креатинина была значительно выше, чем у детей, госпитализированных до пандемии (P < 0,05). У младенцев младше 6 месяцев, рожденных во время пандемии, задержка роста и истощение были значительно выше, чем у младенцев в период до COVID-19 (P < 0,05). Риск смерти у младенцев младше 6 месяцев во время пандемии был выше (отношение шансов: 1,66; 95%-й ДИ: 0,95–2,92).

**Вывод:**

Во время пандемии в больницу поступали дети с более тяжелым течением заболевания и более выраженным недоеданием. Необходимо предпринимать усилия в целях уменьшения негативного воздействия пандемии на здоровье и благополучие детей.

## Introduction

The growing global incidence of coronavirus disease 2019 (COVID-19) caused by severe acute respiratory syndrome coronavirus 2 (SARS-CoV-2) has led the World Health Organization (WHO) to acknowledge COVID-19 as a public health emergency.^1^ The unprecedented global, social and economic crisis created by the pandemic has brought unpredictable threats to the nutritional status and survival of young children in low- and middle-income countries.^2^ Childhood malnutrition, including wasting, is expected to increase as a result of sudden decreases in household incomes, food insecurity and disruption of the health-care system.^3^ Before the start of the COVID-19 pandemic, 47 million children younger than 5 years in the world were wasted.^4^ Estimates suggest that during the first year of the pandemic, a further 6.7 million children will have suffered from wasting and an additional 10 000 children will have died.^2^ The COVID-19 pandemic is predicted to result in a reduction in health service coverage of essential pregnancy and newborn care in 132 low- and middle-income countries.^5^

Recent research has shown that COVID-19 will continue to disrupt health and economic indicators worldwide, including progress in maternal and child nutrition. Based on the MIRAGRODEP model, a multicountry, multisector world economy equilibrium model, the COVID-19 pandemic will result in a 20% increase in global poverty.^6^ To determine how much the COVID-19 pandemic will affect the health and development of children requires an evidence-based approach.^7^

Currently, we lack evidence on the influence of COVID-19 on morbidity and nutrition in children younger than 5 years, including infants younger than 6 months and children born during the pandemic. These infants not only have the greatest growth velocity and a unique physiology,^8^ but are also vulnerable to a lack of adequate nutrition.^9^ In our hospital we observed that infants admitted to the hospital during the COVID-19 pandemic were sicker than infants admitted before the pandemic.

In this study, we aimed to assess changes in the health and nutritional status of children younger than 5 years and infants younger than 6 months admitted to hospital during the COVID-19 pandemic compared with the health and nutritional status of children of the same age admitted in the pre-COVID-19 period. In addition, we aimed to assess the severity of illness of children who were born and admitted to hospital during the COVID pandemic.

## Methods

### Study site

The Dhaka Hospital of the International Centre for Diarrhoeal Disease Research, Bangladesh (icddr,b) is the world’s largest diarrhoeal disease treatment facility^10^ and provides free treatment to about 200 000 patients annually. This hospital has advanced laboratory facilities capable of performing all types of routinely prescribed diagnostic tests. All units of the hospital were in operation during the COVID-19 pandemic. On admission to the hospital, children are assessed by triage nurses for dehydration and co-morbidities. Children without dehydration or co-morbidities are moved to the outpatient department, and those with dehydration but without co-morbidities are shifted to the short-stay unit. Children with dehydration and co-morbidities, including electrolyte imbalance, enteric fever or severe malnutrition, are transferred to inpatient wards, which include the longer stay unit and the intensive care unit. The intensive care unit is equipped with invasive and non-invasive ventilation and other facilities for management of critically ill patients.

### Study design and data collection

This study was a cross-sectional study of the patient records of children admitted to the hospital from March 2019–February 2021. We collected data on sociodemographic characteristics (age, sex, type of delivery, immunization status and breastfeeding), anthropometric indices (stunting, wasting, and severe acute malnutrition), clinical characteristics (acute diarrhoea, dehydration, fever, convulsions, pneumonia, severe pneumonia, sepsis, severe sepsis or septic shock, hospital-acquired infection and death) and biochemical measures (hypernatraemia, hyponatraemia, hyperkalaemia, hypokalaemia and raised creatinine) of the children.

We assessed nutritional status based on *z*-scores, calculated according to the WHO 2006 growth standards.^11^ We defined severe acute malnutrition in children of 0–59 months as weight-for-length or -height *z*-score < −3 standard deviations (SD) or by the presence of bilateral pedal oedema, irrespective of anthropometric indicators. We defined underweight as weight-for-age *z*-score < −2 SD, and stunting as length- or height-for-age *z*-score < −2 SD.

**Fig. 1:**
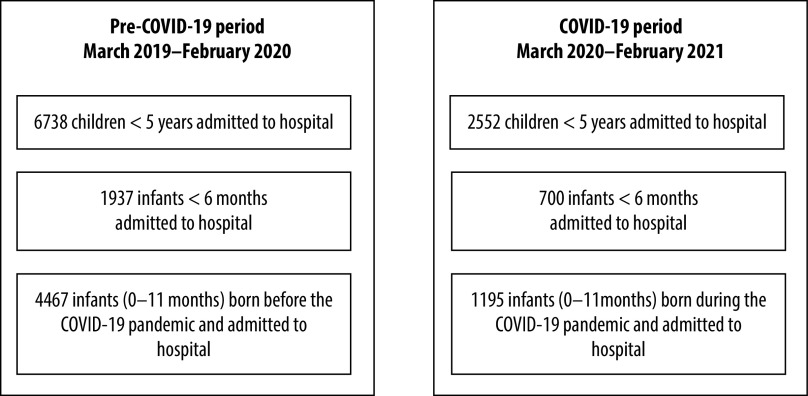
Children included in the study in the COVID-19 and pre-COVID-19 periods by age category, Bangladesh, March 2019–February 2021

We categorized the children into two time periods: children admitted in the pre-COVID-19 period (March 2019–February 2020) and children admitted in the COVID-19 period (March 2020–February 2021). We compared the characteristics of the children in the two periods. Fig. 1 shows the numbers of children and infants included in the study in the pre-COVID-19 period and COVID-19 period.

COVID-19: coronavirus disease 2019.

### Statistical analysis

We report categorical variables as numbers and percentages and normally distributed quantitative variables as mean and SD. We used the χ^2^ test to compare categorical variables between the groups. We considered the COVID-19 pandemic period as the independent variable and the dependent variables as: anthropometric indices, acute watery and/or invasive diarrhoea, dehydration, fever, convulsions, pneumonia, severe pneumonia, sepsis, severe sepsis or septic shock, hospital-acquired infection, death and abnormal electrolyte status.

We used logistic regression analysis to assess the strength of associations between the COVID-19 pandemic and anthropometric indices, morbidities and mortality after adjusting for potential confounders (age and sex), reported as odds ratios (ORs) and 95% confidence intervals (CIs). We considered *P* < 0.05 statistically significant.

We used Stata version 13.0 IC (StataCorp, College Station, United States of America) for all analyses.

### Ethical considerations

We retrieved data from the electronic database of patient records of the hospital. The institutional review board of the International Centre for Diarrhoeal Disease Research, Bangladesh approved the study. We de-identified data for analysis and publication.

## Results

**Fig. 2:**
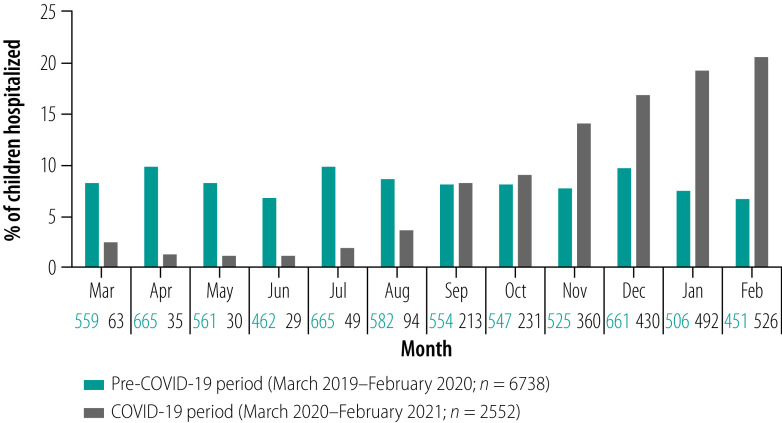
Monthly hospital admissions of children younger than 5 years in the COVID-19 and pre-COVID-19 periods, Bangladesh, March 2019–February 2021

In total, 6738 and 2552 children younger than 5 years were admitted to inpatient wards during the pre-COVID-19 and COVID-19 periods, respectively. From March to August 2020, the hospital admission rate was significantly lower compared with the same time of the year in 2019. This decrease was due to the movement restrictions imposed during this period. From September 2020, with the removal of movement restrictions, hospital admissions gradually increased and in March 2021, admissions were three times higher than admissions in March 2019. Fig. 2 shows the monthly hospital admissions in the pre-COVID-19 and COVID-19 periods. As data were collected from hospital records, information was missing for some variables. We calculated percentage value on the basis of available records.

COVID-19: coronavirus disease 2019.

Note: The percentage is of total admissions for each period. Data are from the Dhaka Hospital of the International Centre for Diarrhoeal Disease Research, Bangladesh.

### Children

Characteristics of the children younger than 5 years admitted to hospital during and before the COVID-19 pandemic are compared in Table 1. Children admitted to the hospital in the pandemic period were significantly younger than those admitted in the pre-pandemic period (11.11 months; SD: 8.54 versus 11.59 months; SD: 9.65; *P* = 0.020). Significantly more children admitted in the COVID-19 period had been delivered by caesarean section than children born before the pandemic (48.87%; 887/1815 versus 45.74%; 1875/4099; *P* = 0.026).

**Table 1:** Characteristics of children admitted to hospital during and before the COVID-19 pandemic by age category, Bangladesh, March 2019–February 2021

Characteristic	Children < 5 years				Infants < 6 months		
	No. (%)^a^		*P*		No. (%)^a^		*P*
	COVID-19 period (*n* = 2552)	Pre-COVID-19 period (*n* = 6738)			COVID-19 period (*n* = 700)	Pre-COVID-19 period (*n* = 1937)	
Age in months, mean (SD)	11.11 (8.54)	11.59 (9.65)	0.020		3.44 (1.48)	3.63 (1.49)	0.004
Sex, female	957 (37.50)	2514 (37.31)	0.866		288 (41.14)	741 (38.26)	0.179
Delivery by caesarean section	887/1815 (48.87)	1875/4099 (45.74)	0.026		279/554 (50.36)	600/1224 (49.02)	0.600
Immunization as per EPI schedule	1712/1903 (89.96)	4079/4591 (88.85)	0.188		456/577 (79.03)	1074/1352 (79.44)	0.839
Exclusively or partially breastfed	1424/2075 (68.63)	3418/5181 (65.97)	0.030		372/632 (58.86)	861/1561 (55.16)	0.113
Stunting^b^	1051/2186 (48.08)	2553/5149 (49.58)	0.239		386/597 (64.66)	883/1512 (58.40)	< 0.001
Wasting^c^	863/1933 (44.65)	1977/4383 (45.11)	0.735		237/486 (48.77)	482/1173 (41.09)	0.004
Severe acute malnutrition^d^	694/2171 (31.97)	1813/5177 (35.02)	0.012		233/589 (39.56)	623/1533 (40.64)	0.650
Acute diarrhoea (watery and/or invasive)	2477/2552 (97.06)	6540/6738 (97.06)	0.999		674/700 (96.29)	1869/1937 (96.49)	0.803
Some or severe dehydration	1489/2552 (58.15)	3499/6657 (52.56)	< 0.001		466/697 (66.86)	1073/1901 (56.44)	< 0.001
History of fever	609/2552 (23.86)	1595/6738 (23.67)	0.846		141/700 (20.14)	388/1937 (20.03)	0.949
Convulsions (during and/or after admission)	146/2552 (5.72)	229/6738 (3.4)	< 0.001		47/700 (6.71)	72/1937 (3.72)	0.001
Pneumonia	376/2552 (14.73)	1102/6738 (16.36)	0.056		151/700 (21.57)	409/1937 (21.12)	0.800
Severe pneumonia	217/2552 (8.50)	700/6738 (10.39)	0.007		91/700 (13.00)	292/1937 (15.07)	0.182
Sepsis	233/2552 (9.13)	287/6738 (4.26)	< 0.001		112/700 (16.00)	119/1937 (6.14)	< 0.001
Severe sepsis or septic shock	65/2552 (2.55)	98/6738 (1.45)	< 0.001		30/700 (4.29)	44/1937 (2.27)	0.006
Hospital-acquired infection	41/2552 (1.61)	125/6738 (1.86)	0.420		21/700 (3.00)	45/1937 (2.32)	0.326
Death	31/2552 (1.21)	83/6738 (1.23)	0.947		20/700 (2.86)	33/1937 (1.70)	0.062
Hypernatraemia^e^	213/1053 (20.23)	223/1520 (14.67)	< 0.001		52/234 (22.22)	72/389(18.51)	0.261
Hyponatraemia^f^	340/1053 (32.29)	682/1520 (44.87)	< 0.001		87/234 (37.18)	165/389 (42.42)	0.197
Hyperkalaemia^g^	89/1053 (8.45)	139/1520 (9.14)	0.543		50/234 (21.37)	75/389 (19.28)	0.529
Hypokalaemia^h^	249/1053 (23.65)	407/1520 (26.78)	0.073		62/234 (26.50)	94/389 (24.16)	0.515
Raised creatinine^i^	200/577 (34.66)	318/1091 (29.15)	0.021		98/169 (57.99)	144/307 (46.91)	0.021

COVID-19: coronavirus disease 2019; SD: standard deviation; EPI: Expanded Programme on Immunization.

^a^ All values are no. (%) unless otherwise stated.

^b^ Length- or height-for-age *z*-score < −2 SD.

^c^ Weight-for-length or -height *z*-score < −2 SD.

^d^ Weight-for-length or -height *z*-score < −3 SD or by the presence of bilateral pedal oedema, irrespective of anthropometric indicators.

^e^ Serum sodium ≥ 150 mmol/L.

^f^ Serum sodium < 135 mmol/L.

^g^ Serum potassium > 5.5 mmol/L.

^h^ Serum potassium < 3.5 mmol/L.

^i^ Serum creatinine > 35 μmol/L for < 12 months and > 65 μmol/L for 12–59 months.

Notes: The COVID-19 period was March 2020–February 2021 and the pre-COVID-19 period was March 2019–February 2020. As data were collected from hospital records, information was missing for some variables. We calculated percentage value on the basis of available records.

In age- and sex-adjusted logistic regression analysis, the odds of dehydration (OR: 1.25; 95% CI: 1.15–1.38), convulsions (OR: 1.74; 95% CI: 1.41–2.16), sepsis (OR: 2.25; 95% CI: 1.88–2.69), severe sepsis or septic shock (OR: 1.76; 95% CI: 1.28–2.41), hypernatraemia (OR: 1.47; 95% CI: 1.19–1.82) and raised creatinine level (OR: 1.25; 95% CI: 1.00–1.57) were significantly higher in children in the COVID-19 period than children in the pre-COVID-19 period (Table 2). The odds of severe acute malnutrition (OR: 0.87; 95% CI: 0.78–0.97), pneumonia (OR: 0.87; 95% CI: 0.77–0.99) and severe pneumonia (OR: 0.79; 95% CI: 0.67–0.93) were significantly lower in the COVID-19 period than the pre-COVID-19 period. The odds of hospital-acquired infection and death were comparable in both periods.

**Table 2:** Risk of illness, death and undernutrition in children admitted to hospital during COVID-19 by age category, Bangladesh, March 2020–February 2021

Variable	aOR^a^ (95% CI)	
	Children < 5 years	Infants < 6 months
**Illness and death**		
Acute diarrhoea (watery and/or invasive)	1.00 (0.76–1.32)	0.93 (0.59–1.48)
Some or severe dehydration	1.25 (1.15–1.38)	1.54 (1.28–1.84)
History of fever	1.02 (0.92–1.14)	1.03 (0.83–1.28)
Convulsions (during and/or after admission)	1.74 (1.41–2.16)	1.89 (1.29–2.76)
Pneumonia	0.87 (0.77–0.99)	1.04 (0.84–1.28)
Severe pneumonia	0.79 (0.67–0.93)	0.84 (0.65–1.08)
Sepsis	2.25 (1.88–2.69)	2.79 (2.11–3.68)
Severe sepsis or septic shock	1.76 (1.28–2.41)	1.85 (1.15–2.98)
Hospital-acquired infection	0.86 (0.60–1.22)	1.27 (0.75–2.16)
Death	0.97 (0.64–1.48)	1.66 (0.95–2.92)
**Anthropometric indices**		
Stunting^b^	0.94 (0.85–1.04)	1.28 (1.05–1.56)
Wasting^c^	1.00 (0.90–1.11)	1.38 (1.11–1.71)
Severe acute malnutrition^d^	0.87 (0.78–0.97)	0.98 (0.80–1.90)
**Electrolyte imbalance**		
Hypernatraemia^e^	1.47 (1.19–1.82)	1.26 (0.84–1.88)
Hyponatraemia^f^	0.59 (0.50–0.70)	0.80 (0.57–1.12)
Hyperkalaemia^g^	0.94 (0.71–1.24)	1.14 (0.76–1.71)
Hypokalaemia^h^	0.85 (0.71–1.02)	1.13 (0.78–1.64)
Raised creatinine^i^	1.25 (1.00–1.57)	1.56 (1.07–2.29)

COVID-19: coronavirus disease 2019; aOR: adjusted odds ratio; CI: confidence interval; SD: standard deviation.

^a^ OR adjusted for age and sex.

^b^ Length- or height-for-age *z-*score < −2 SD.

^c^ Weight-for-length or -height *z*-score < −2 SD.

^d^ Weight-for-length or -height *z*-score < −3 SD or by the presence of bilateral pedal oedema, irrespective of anthropometric indicators.

^e^ Serum sodium ≥ 150 mmol/L.

^f^ Serum sodium < 135 mmol/L.

^g^ Serum potassium > 5.5 mmol/L.

^h^ Serum potassium < 3.5 mmol/L.

^i^ Serum creatinine > 35 μmol/L for < 12 months and > 65 μmol/L for 12–59 months.

Note: Reference categories are children admitted to hospital before the COVID-19 pandemic, March 2019–February 2020.

### Infants

During the COVID-19 period, 700 infants younger than 6 months were admitted to hospital, which was lower than the 1937 infants admitted in the pre-COVID-19 period (Table 1). Infants in the COVID-19 period were significantly younger than those admitted in the pre-COVID-19 period (3.44 months; SD: 1.48 versus 3.63 months; SD: 1.49; *P* = 0.004). The proportion of infants delivered by caesarean section was comparable between the two periods, 50.36% (279/554) for the COVID-19 period and 49.02% (600/1224) for the pre-COVID-19 period. Breastfeeding and immunization in these young infants were comparable in the pre-COVID-19 period and COVID-19 period.

In age- and sex-adjusted logistic regression analysis, the incidence of wasting (OR: 1.38; 95% CI: 1.11–1.71) and stunting (OR: 1.28; 95% CI: 1.05–1.56) in infants < 6 months was higher in the COVID-19 period than the pre-COVID-19 period (Table 2). Infants < 6 months admitted to hospital in the COVID-19 period had significantly higher odds of dehydration (OR: 1.54; 95% CI: 1.28–1.84), convulsions (OR: 1.89; 95% CI: 1.29–2.76), sepsis (OR: 2.79; 95% CI: 2.11–3.68), severe sepsis or septic shock (OR: 1.85; 95% CI: 1.15–2.98) and raised creatinine (OR: 1.56; 95% CI: 1.07–2.29) than infants admitted in the pre-COVID-19 period (Table 2). The odds of death were also higher in infants in the COVID-19 period than in the pre-COVID-19 period, although the difference was not statistically significant (OR: 1.66; 95% CI: 0.95–2.92; Table 2).

### Infants aged 0–11 months

Of the children under 5 years admitted to hospital, 1195 were born in the COVID-19 period and were aged 0 to 11 months. We compared these infants with 4467 infants of the same age group (0–11 months) admitted in the pre-COVID-19-period (Table 3).

**Table 3:** Characteristics of infants 0–11 months born and admitted to hospital during and before the COVID-19 pandemic, Bangladesh, March 2019–February 2021

Characteristic	No. (%)^a^			*P*
	All (*n* = 5662)	COVID-19 period (*n* = 1195)	Pre-COVID-19 period (*n* = 4467)	
Age in months, mean (SD)	6.29 (2.94)	5.59 (2.80)	6.48 (2.95)	< 0.001
Sex, female	2103/5662 (37.14)	457/1195 (38.24)	1646/4467 (36.85)	0.375
Delivery by caesarean section	1756/3669 (47.86)	450/895 (50.28)	1306/2774 (47.08)	0.096
Immunization as per EPI schedule	3475/4012 (86.62)	786/923 (85.16)	2689/3089 (87.05)	0.138
Exclusive or partial breastfeeding on admission	2920/4509 (64.76)	674/1008 (66.87)	2246/3501 (64.15)	0.112
Stunting^b^	2323/4518 (51.42)	575/1 044 (55.08)	1748/3474 (50.32)	0.007
Wasting^c^	1522/3 694 (41.20)	400/891 (44.89)	1122/2803 (40.03)	0.010
Severe acute malnutrition^d^	1642/4548 (36.10)	366/1041 (35.16)	1276/3507 (36.38)	0.470
Acute diarrhoea (watery and/or invasive)	5460/5662 (96.43)	1159/1195 (96.99)	4301/4467 (96.28)	0.244
Some or severe dehydration	3033/5600 (54.16)	740/1191 (62.13)	2293/4409 (52.01)	< 0.001
History of fever	1254/5662 (22.15)	267/1195 (22.34)	987/4467 (22.10)	0.855
Convulsions (during and/or after admission)	219/5662 (3.87)	70/1195 (5.86)	149/4467 (3.34)	< 0.001
Pneumonia	1097/5662 (19.37)	225/1195 (18.83)	872/4467 (19.52)	0.591
Severe pneumonia	683/5662 (12.06)	126/1195 (10.54)	557/4467 (12.47)	0.070
Sepsis	348/5662 (6.15)	134/1195 (11.21)	214/4467 (4.79)	< 0.001
Severe sepsis or septic shock	110/5662 (1.94)	32/1195 (2.68)	78/4467 (1.75)	0.038
Hospital-acquired infection	129/5662 (2.28)	22/1195 (1.84)	107/4467 (2.40)	0.254
Death	82/5662 (1.45)	20/1195 (1.67)	62/4467 (1.39)	0.463
Hypernatraemia^e^	294/1 419 (20.72)	110/447 (24.61)	184/ 972 (18.93)	0.014
Hyponatraemia^f^	499/1 419 (35.17)	116/447 (25.95)	383/972 (39.40)	< 0.001
Hyperkalaemia^g^	175/1419 (12.33)	56/ 447 (12.53)	119/972 (12.24)	0.879
Hypokalaemia^h^	349/1 419 (24.59)	102/447 (22.82)	247/972 (25.41)	0.292
Raised creatinine^i^	405/977 (41.45)	128/258 (49.61)	277/719 (38.53)	0.002

COVID-19: coronavirus disease 2019; SD: standard deviation; EPI: Expanded Programme on Immunization.

^a^ All values are no. (%) unless otherwise stated.

^b^ Length-for-age *z*-score < −2 SD

^c^ Weight-for-length *z*-score < −2 SD

^d^ Weight-for-length *z*-score < −3 SD or by the presence of bilateral pedal oedema, irrespective of anthropometric indicators.

^e^ Serum sodium ≥ 150 mmol/L.

^f^ Serum sodium < 135 mmol/L/.

^g^ Serum potassium > 5.5 mmol/L.

^h^ Serum potassium < 3.5 mmol/L.

^i^ Serum creatinine > 35 μmol/L for < 12 months and > 65 μmol/L for 12–59 months.

Notes: The COVID-19 period was March 2020–February 2021 and the pre-COVID-19 period was March 2019–February 2020. As data were collected from hospital records, information was missing for some variables. We calculated percentage value on the basis of available records.

Infants 0–11 months admitted during the COVID-19 period were significantly younger at admittance than infants of the same age admitted in the pre-COVID-19 period (5.59 months; SD: 2.80 versus 6.48 months; SD: 2.95; *P* < 0.001). Significantly greater proportions of infants 0–11 months admitted in the COVID-19 period than those admitted in the pre-COVID-19 period were stunted and wasted, dehydrated, had convulsions, had sepsis and severe sepsis, and had hypernatraemia and raised creatinine (*P* < 0.05) (Table 3). In logistic regression analysis, with adjustment for age and sex, infants born during the COVID-19 period compared with those born in the pre-COVID-19 period had higher odds of: dehydration (OR: 1.43; 95% CI: 1.26–1.64), convulsions (OR: 1.74; 95% CI: 1.30–2.34), sepsis (OR: 2.21; 95% CI: 1.76–2.78), wasting (OR: 1.21; 95% CI: 1.03–1.41), hypernatraemia (OR: 1.43; 95% CI: 1.09–1.88) and raised creatinine (OR: 1.38; 95% CI: 1.03–1.86; Table 4). However, the odds of severe pneumonia (OR: 0.77; 95% CI: 0.62–0.94) and hyponatraemia (OR: 0.50; 95% CI: 0.39–0.64) were significantly lower in the infants born and admitted in the COVID-19 period. The likelihood of death was not significantly higher in the infants in the COVID-19 period compared with infants in the pre-COVID period (OR: 1.12; 95% CI: 0.67–1.86).

**Table 4:** Risk of illness, death and undernutrition in infants born during the COVID-19 pandemic admitted to hospital, Bangladesh, March 2020–February 2021

Variable	aOR^a^ (95% CI)
**Illness and death**	
Acute diarrhoea (watery and/or invasive)	1.24 (0.86–1.79)
Some or severe dehydration	1.43 (1.26–1.64)
History of fever	1.06 (0.91–1.24)
Convulsions (during and/or after admission)	1.74 (1.30–2.34)
Pneumonia	0.92 (0.78–1.08)
Severe pneumonia	0.77 (0.62–0.94)
Sepsis	2.21 (1.76–2.78)
Severe sepsis or septic shock	1.37 (0.90–2.09)
Hospital-acquired infection	0.75 (0.47–1.19)
Death	1.12 (0.67–1.86)
**Anthropometric indices**	
Stunting^b^	1.10 (0.96–1.27)
Wasting^c^	1.21 (1.03–1.41)
Severe acute malnutrition^d^	0.91 (0.79–1.05)
**Electrolyte imbalance**	
Hypernatraemia^e^	1.43 (1.09–1.88)
Hyponatraemia^f^	0.50 (0.39–0.64)
Hyperkalaemia^g^	0.92 (0.65–1.30)
Hypokalaemia^h^	0.85 (0.65–1.11)
Raised creatinine^i^	1.38 (1.03–1.86)

COVID-19: coronavirus disease 2019; aOR: adjusted odds ratio; CI: confidence interval; SD: standard deviation.

^a^ OR adjusted for age and sex.

^b^ Length-for-age *z*-score < −2 SD.

^c^ Weight-for-length *z*-score < −2 SD.

^d^ Weight-for-length *z*-score < −3 SD or presence of bilateral pedal oedema, irrespective of anthropometric indicators.

^e^ Serum sodium ≥ 150 mmol/L.

^f^ Serum sodium < 135 mmol/L.

^g^ Serum potassium > 5.5 mmol/L.

^h^ Serum potassium < 3.5 mmol/L.

^i^ Serum creatinine > 35 μmol/L for < 12 months and > 65 μmol/L for 12–59 months.

Note: Reference categories are infants born and admitted to hospital before the COVID-19 pandemic, March 2019–February 2020.

## Discussion

Our study findings show that COVID-19 is already affecting the health of children, especially young infants, with a significantly higher percentage of critically ill children being treated at our facility in the COVID-19 period compared with during the pre-COVID-19 period. Moreover, compared to the pre-COVID-19 period, these infants had a higher proportion of deaths, although the difference was not statistically significant.

We found a marked reduction in admission of children between April 2020 and August 2020. After the detection of the first COVID-19 case in Bangladesh,^12^ the country imposed a complete stay-at-home order for 2 weeks from 26 March 2020.^13^ The stay-at-home order was extended to 30 May 2020,^14^ and was then followed by restrictions on movement and limited business hours.^15^ Restrictions on movement were officially lifted on 1 September 2020. As a result, admission of children to hospital gradually increased from September 2020 onwards in the COVID-19 period. Admissions of infants in February 2021 were three times higher than in the corresponding pre-COVID-19 period of 2019.

An inpatient survey in an Australian paediatric hospital suggested that more than one third of parents delayed medical care visits for their children from fear of COVID-19.^16^ Other studies from different parts of the world have also reported such delays.^17^^,^^18^

The COVID-19 pandemic is likely to have affected health-care systems in many ways. The government of Bangladesh is concerned about reduced coverage and quality of maternal and child health services, but little evidence is available on health service provision, use or adaptation during COVID-19.^19^ The most important factors identified for the disruption of the health system are disturbances to the livelihood of people from the stay-at-home order and related restriction measures, and the lack of protection measures for health-care workers.^20^ A recent study documented a decline in visits to health facilities for regular antenatal care during April and May 2020 in Bangladesh compared with the same months in 2019.^20^ Likewise, visits to family planning clinics and child immunization centres also declined.^20^ Our findings on children born during the COVID-19 period also showed lower proportions of immunization in the COVID-19 period (85.16%; 786/923 versus 87.05%; 2689/3089), although the difference was not statically significant. Due to the effect of movement restrictions in Bangladesh, routine immunizations have been severely disrupted, with parents being reluctant to take their children to health-care facilities for routine care. Despite the continuation of routine immunization in the country, many catch-up drives and vaccine campaigns were suspended and the transport of vaccines to different parts of the country is still challenging. To combat vaccine-preventable diseases, the Directorate-General of Health Services in Bangladesh issued guidelines to continue routine immunization during the COVID-19 pandemic, in line with global and regional advisories from the United Nations Children’s Fund and WHO.^21^

A study on contextual factors influencing maternal, neonatal and child health care in Bangladesh, Nigeria and South Africa also reported that during the initial period of the pandemic, the use of health facilities for normal deliveries in Bangladesh decreased, which was attributable to more home births.^20^ However, we observed a higher proportion of children delivered by caesarean section during the pandemic period than those born during the pre-COVID-19 period, although the difference was not statistically significant. Our analysis also showed that during COVID-19, breastfeeding improved in children younger than 5 years compared with the pre-COVID-19 period. We had no data on the duration of exclusive breastfeeding, but the use of breast-milk substitutes in Bangladesh reportedly increased during COVID-19.^21^

Health services for children younger than 5 years have declined significantly in Bangladesh due to the COVID-19 pandemic.^21^ The use of health services for children of this age group in March 2020 fell by 25% compared with service use in March 2019.^21^ Research on the indirect effect of COVID-19 in 118 low- and middle-income countries, based on the worst of three scenarios, suggested that even a minor disruption to health-care services could increase childhood wasting by 10%.^22^ Our study supports these findings. Among the young infants, wasting and stunting was significantly higher in the pandemic period and a greater proportion of children born during the pandemic were wasted compared with children in the same age group born in the pre-COVID-19 period.

National nutrition strategies had been implemented in Bangladesh to reduce the rates of maternal and child undernutrition, but problems with coordination resulted in poor delivery of nutrition services.^23^ The uptake of maternal and newborn health services decreased by about 19% during the pandemic.^21^ Various initiatives have been adopted by the public and private sectors to deal with the adverse effects of the COVID-19 pandemic on the health-care system.^19^ Development partners including local nongovernmental organizations supported the National Nutrition Services of Bangladesh to develop the national guidelines on continuing essential nutrition services during the COVID-19 pandemic.^19^

It was anticipated that disruption to essential services may result in a 37% increase in child mortality in Bangladesh by 2021.^21^ In our analysis, although admissions of children to hospital fell in the pandemic period, a significantly higher percentage of the children admitted were critically ill with dehydration, sepsis, convulsions and electrolyte imbalances. During the pandemic there was a higher mortality than during the pre-pandemic period, although the difference was not statistically significant. This reduction in hospital admissions has raised concerns about late presentation of critically ill children.^24^ The higher incidence of severe illness among children in the COVID-19 period than in the previous year may be a result of delays by caregivers in bringing their children to hospital because of strict movement restrictions, the nationwide stay-at-home order and the fear of contracting COVID-19. In an electronic survey on 24 April 2020 of 752 British paediatricians working in emergency departments and paediatric assessment units, 241 (32.05%) reported that they had observed delayed presentations. Sepsis was the second most common condition found in the children who presented late to the hospital.^25^ While COVID-19 and paediatric inflammatory multisystem syndrome temporally associated with SARS-CoV-2 or multisystem inflammatory syndrome in children have attracted particular attention, we should not forget non-COVID-19 sepsis, the incidence of which is still higher in children than sepsis associated with COVID-19.^26^ Our findings concur with this observation as we did not encounter any COVID-related illness in children in our hospital. In 2005 in the United States, there were 75 255 paediatric hospitalizations involving severe sepsis indicating that sepsis was common before COVID-19.^27^

With regard to electrolyte imbalance, we observed that hypernatraemia and raised serum creatinine levels were the most common imbalances in the COVID-19 period. Hypernatraemia in children with diarrhoea has several causes. In our study, hypernatraemia was probably a result of delayed presentation of the children, because caregivers tried at home to treat the child with oral rehydration salts before coming to the health facility; these preparations may have been incorrectly made up or given too often or in too large a volume.^28^ Another study reported that children with hypernatraemia were more likely to have convulsions^29^ than children without hypernatraemia. This observation could explain why more children in our study had hypernatraemia in the COVID-19 period than the pre-COVID-19 period. Hyponatraemia is common in people with cholera because of the high loss of sodium in the stool. This condition is more common in invasive diarrhoea because of the syndrome of inappropriate antidiuretic hormone secretion.^30^ During the COVID-19 period, the higher prevalence of handwashing might be associated with the lower incidence of cholera and invasive diarrhoea compared with the pre-COVID-19 period and hence the lower incidence of hyponatraemia.

Our study had some limitations. We used objective criteria from a guideline on surviving sepsis and identification of sepsis, severe sepsis and septic shock;^31^ even so, there might have been subjective bias in identifying children with sepsis. We lacked data on maternal stress, maternal COVID-19 status and non-COVID infections and inflammation during and after pregnancy, as well as data on potential socioeconomic crises faced by the families during this pandemic. Although our study took place in the largest diarrhoeal disease facility in the world,^10^ these data, together with nationwide data on disease severity and deaths among such young infants during the COVID-19 pandemic would have enhanced the reliability of our observations.

We believe that more effective means of risk assessment, the development of a multisectoral management taskforce and appropriate governance for the proper management of the health sector to ensure basic support for patients, particularly for vulnerable groups, are needed to reduce the adverse effects of the COVID-19 pandemic on the health and well-being of children, especially that of young infants.
